# Corticosteroid induced avascular necrosis and COVID-19: The drug dilemma

**DOI:** 10.3126/nje.v11i3.39309

**Published:** 2021-09-30

**Authors:** Indrajit Banerjee, Jared Robinson, Brijesh Sathian

**Affiliations:** 1, 2 Sir Seewoosagur Ramgoolam Medical College, Belle Rive, Mauritius; 3 Geriatric and long term care Department, Rumailah Hospital, Hamad Medical Corporation, Doha, Qatar

## Background

The SARS-CoV-2 pandemic has swiftly and firmly implanted itself within our communities and countries. Multiple countries have faced numerous waves of the virus and have paid the price with death counts totalling in the hundreds of thousands, and globally now millions. The novel nature and rapidity of the SARS-CoV-2 spread throughout the globe has resulted in health institutions finding themselves in a quagmire as they battle with the lack of the necessary equipment but more importantly battle the lack of an established and universally accepted treatment protocol for the COVID-19 infection [1].

The drugs thus used throughout the progression of the pandemic have varied and waivered as more research and data for better treatment of the infection has become available. Corticosteroids are however, one such group of drugs that are almost always a constant among most treatment regimens and protocols. The off label use and implementation of various regimens and high doses of certain drugs have led to some deleterious adverse effects [2].

### Corticosteroids in COVID-19

Corticosteroids and immunosuppressants of such a nature have long been used in viral infections and have been a mainstay in the treatment protocol and regimens used in COVID-19 patients. Popular corticosteroid drugs and therapies that are being prescribed in patients suffering from COVID-19 are dexamethasone, methylprednisolone and or hydrocortisone with IV (intravenous) and/or oral administration. The use of such high doses of corticosteroids have shown very positive results and have been lifesaving in many cases [3]. The basis of the use of such corticosteroid drugs in patients suffering from COVID-19 is the immunosuppressant nature of the drugs. A life-threatening complication and manifestation seen in severely ill patients suffering from COVID-19 is the cytokine release syndrome (cytokine storm) which results in the most severe form of the coronavirus disease (SARS-CoV-19) and manifests itself via ARDS (acute respiratory distress syndrome), disseminated intravascular coagulopathies (DIC) and, or multiple organ failure. The corticosteroids thereby are used to try and prevent such a severe manifestation and complication of the viral infection, however as in the case with all pharmacotherapies adverse effects are par for the course and corticosteroids are no exception to this rule [4,5].

### Complications of Corticosteroid therapy

Corticosteroid therapy has a multitude of side effects and they vary dependant on the dosing, duration and potency of the particular species being prescribed. When used in short durations at high doses the side effects may vary and include hypertension, electrolyte abnormalities, cutaneous effects, pancreatitis, hematological dyscrasias, hyperglycemia, neuropsychologic and immunological adverse effects [6]. In the long-term use of such corticosteroid drugs the adverse effects may range from moderate to more severe and potentially life-threatening side effects such as hypothalamic-pituitary-adrenal axis suppression, reduction in linear growth velocity (stunting of linear growth), drug induced diabetes, osteoporosis, Cushing syndrome, muscle wasting, peripheral fat mobilization, hirsutism, sleep disturbances and poor wound healing [6,7].

Reports have surfaced with new side effects developing in patients post recovery from COVID-19 infections. Severe systemic mucormycotic infections causing orbital compartment syndrome and severe multiorgan infections have been partially attributed to the extensive use of systemic corticosteroids in the treatment of the initial COVID-19 infection. A factor further superadded to the complications experienced is the fact that the majority of patients being treated with corticosteroids have pre-existing conditions and severe comorbidities. The nature of these pre-existing conditions are often exacerbated and accelerated via the use of the lifesaving steroid treatment. A textbook example being the reduced and worsened glycaemic control in diabetic patients whom are treated using corticosteroids [8,9].

As in the case of mucormycotic infections and their relationship with the immunosuppressive nature of corticosteroid therapy used in the treatment of COVID-19; a rise in cases of avascular necrosis of the femoral head is being reported and is being attributed to the sustained and aggressive corticosteroid therapy being implemented [10,11].

### Steroid induced avascular necrosis of the femoral head (SANFH)

The severe and life-threatening nature of the COVID-19 infection, the ARDS (acute respiratory distress syndrome) as well as the cytokine storm induced by the infection, commands lifesaving high doses of steroid therapy. As in all pharmacological therapies adverse effects are present. One such adverse effect which is being reported is corticosteroid induced avascular necrosis of the femoral head/ osteonecrosis of the femoral head (ONFH) [11].

The mechanism by which steroids induce avascular necrosis of the femoral head is underpinned by the collective actions of the drug therapy. It must be noted that AVN principally affects the femoral head and most commonly the anterolateral aspect thereof as it is the crux of weight bearing. Corticosteroids induce fat mobilization and this thus innately enhances the likelihood of fat emboli developing from the liver to occlude minor blood vessels in the femur, this thereby compromises the microvascular environment. Superadded to this the steroid therapy disrupts calcium metabolism and homeostasis which induces hypertrophy in the intramedullary fat cells, Gaucher cells and inflammatory cells; whilst increasing the activity of osteoclasts, thus increasing bone resorption and decreasing calcium uptake and deposition; ultimately leading to an insufficiency in the trabecular and cortical bone. This insufficiency thus equates to an increased intraosseous pressure which impedes intramedullary circulation and results in avascular necrosis [12-14].

Agarwala et al, conducted a study on three cases whom had recovered from COVID-19 after being treated with corticosteroid therapy and subsequently developed AVN. The patients were found to be prescribed a mean dose equivalent to 758mg of prednisolone. The patients were subsequently symptomatic with bilateral femoral hip pain. The great importance of this study is that the patients were diagnosed with AVN after receiving a dosage much lower than the 2000mg equivalent ceiling which current guidelines dictate to avoid AVN. Superadded to this; it was noted that the patients presented with the features of AVN at a mean of 58 days after their initial COVID-19 diagnosis and treatment. This is in contrast to the current literature which states that AVN takes 6 months to 1 year to develop post corticosteroid therapy [15,17].

It has been long established that steroids induced AVN, however reports of AVN developing rapidly in COVID-19 patients post treatment where specific treatment guidelines to avoid steroid induced AVN have been adhered to suggest that the COVID-19 infection itself may also be instrumental in the development of AVN and that steroid use is not the sole benefactor to inducing AVN post steroid therapy [15,16].

### Prevention

Current guidelines suggest that a cumulative ceiling dose of 2000mg of prednisolone or its equivalent should not be breached in order to prevent the development of AVN, however as seen in the research conducted by Agarwal et al it is evident that AVN is being induced in patients treated far below the 2000mg ceiling.[17] It is therefore poignant that the minimum effective dosing of steroids is implemented and prescribed. It is suggested that the simultaneous dosing of the bisphosphonate group of drugs and in particular ibandronate at a dose of 2mg every three months is efficacious and well-tolerated to aid in the prevention of steroidal induced side effects. It is also noted that in patients with contraindications to bisphosphonate therapy, that calcitonin therapy can be implemented. It is suggested that patients receiving a dose of a prednisone equivalent of 5 mg/day or higher are at risk and bisphosphonate therapy should be initiated with recurrent and frequent bone density surveillance. Early stages of AVN may be managed with bisphosphonate therapy and should be implemented to halt the progression of the pathology so as to avoid surgical intervention such as cortical decompression surgery [18-20].

## Conclusion

The use of steroid therapy in COVID-19 is invaluable and lifesaving in nature. The adverse effects of such steroid therapy are however profound and well established. It is evident that avascular necrosis is directly caused by high dose steroid therapy, however the case reports have very clearly indicated that the rapid onset of AVN post recovery from the COVID-19 infection cannot be solely attributed to steroid therapy and that another benefactor induced by the COVID-19 infection is at play. It is thus vital for treating physicians to take cognisance of this adverse effect post recovery and therefore should ensure that prophylactic bisphosphonate therapy is initiated timeously and congruently.
